# PRMT1 Is a Novel Regulator of Epithelial-Mesenchymal-Transition in Non-small Cell Lung Cancer*

**DOI:** 10.1074/jbc.M114.636050

**Published:** 2015-04-06

**Authors:** Sreedevi Avasarala, Michelle Van Scoyk, Manoj Kumar Karuppusamy Rathinam, Sereke Zerayesus, Xiangmin Zhao, Wei Zhang, Melissa R. Pergande, Jeffrey A. Borgia, James DeGregori, J. David Port, Robert A. Winn, Rama Kamesh Bikkavilli

**Affiliations:** From the ‡ Division of Pulmonary, Critical Care, Sleep and Allergy, and; the § Department of Pediatrics, University of Illinois at Chicago, Chicago, Illinois 60612,; the ¶ Department of Pathology and Department of Biochemistry, Rush University Medical Center, Chicago, Illinois 60612, and; the ‖ Department of Biochemistry and Molecular Genetics, and; the ** Departments of Medicine, Cardiology and Pharmacology, University of Colorado Anschutz Medical Campus, Aurora, Colorado 80045

**Keywords:** cadherin-1 (CDH1) (epithelial cadherin) (E-cadherin), epithelial-mesenchymal transition (EMT), metastasis, N-cadherin, protein methylation, Prmt1, Twist1

## Abstract

Protein arginine methyl transferase 1 (PRMT1) was shown to be up-regulated in cancers and important for cancer cell proliferation. However, the role of PRMT1 in lung cancer progression and metastasis remains incompletely understood. In the present study, we show that PRMT1 is an important regulator of epithelial-mesenchymal transition (EMT), cancer cell migration, and invasion, which are essential processes during cancer progression, and metastasis. Additionally, we have identified Twist1, a basic helix-loop-helix transcription factor and a well-known E-cadherin repressor, as a novel PRMT1 substrate. Taken together, we show that PRMT1 is a novel regulator of EMT and arginine 34 (Arg-34) methylation of Twist1 as a unique “methyl arginine mark” for active E-cadherin repression. Therefore, targeting PRMT1-mediated Twist1 methylation might represent a novel strategy for developing new anti-invasive/anti-metastatic drugs. Moreover, methylated Twist1 (Arg-34), as such, could also emerge as a potential important biomarker for lung cancer.

## Teaser

**Background:** PRMT1 is up-regulated in lung cancer.

**Results:** PRMT1 is a novel regulator of EMT and Twist1 is a new PRMT1 substrate.

**Conclusion:** PRMT1-methylation of Twist1 is required for active E-cadherin repression.

## Introduction

Lung cancer is the deadliest of all the cancers. High mortalities in lung cancer patients can be partially attributed to (a) difficulties in detecting the disease early, and (b) resistance to currently available standard therapies. Hence, there is a large need for the development of novel strategies to combat this deadly disease. Many cancers display genetic and epigenetic alterations, which strongly contribute to cellular transformation via chromatin remodeling, histone modification, and gene expression; all of these contribute to the gene silencing of tumor suppressors, and the gain of oncogenes. While DNA methylation and chromatin remodeling are considered to be major epigenetic regulators, emerging data implicate protein arginine methylation (a post-translational modification) as a novel epigenetic regulator in cancer (1, 2).

Although protein methylation was first described in 1968 (3), researchers only began to appreciate the importance of this post-translational modification after the cloning of protein arginine methyl transferase 1 (PRMT1)^2^ in 1996 (4). Protein arginine methylation is one of the most abundant post-translational modifications, with about 2% of arginine residues in proteins of nuclear extracts being dimethylated (5). Arginine is a positively charged amino acid and the nitrogen/s of arginine within the protein can be post-translationally modified via the addition of a methyl group, a process known as arginine methylation, and catalyzed by a class of enzymes, referred to as protein arginine methyl transferases (PRMTs). Furthermore, arginines can either be monomethylated or dimethylated and the latter can be either symmetric or asymmetric depending on the type of protein methyl transferase catalyzing the process (6–8). There are a total of ten mammalian PRMTs identified to date, of which eight have been shown to catalyze methylation (6, 9). Based on the end products produced, PRMTs are classified into several families that include, type I family of enzymes (*i.e.* PRMT1, PRMT3, PRMT4/CARM1, PRMT6, PRMT8), type II enzymes (*i.e.* PRMT5, PRMT7, PRMT10), and type IV (*i.e.* PRMT2) enzymes (6, 9).

Proteins that harbor arginine-glycine-rich motifs (RG) are often targets for PRMT-mediated methylation. While histones constitute a class of well-defined PRMT substrates, there is an increasing list of non-histone PRMT substrates, which includes tumor suppressors (*e.g.* p53), RNA-binding proteins (*e.g.* KSRP, G3BP1, G3BP2), transcription factors (*e.g.* FOXO), and protein translation machinery (*e.g.* PABP1, (10–15)).

PRMT-mediated methylation regulates many essential cell functions mainly through the modulation of protein function, gene expression, and/or cellular signaling. In general, arginine methylation of proteins will have a positive or negative impact on the interactions with other molecules, which could be either other proteins or nucleic acids (DNA or RNA). These altered interactions act as “molecular switches,” which affect either the sub-cellular localization of proteins and/or the stability of protein, or RNA; ultimately affecting either gene expression or cellular signaling.

Deregulation of PRMT expression, predominantly up-regulation, has been attributed to several cancers (1, 2, 16, 17). Particularly, PRMT1 and PRMT6, which catalyze asymmetric dimethylarginine formation, were shown to be significantly up-regulated in lung cancer compared with adjacent normal tissue (1, 2, 16, 17). Furthermore, PRMT1 and PRMT6 have also been shown to regulate cancer cell proliferation (16). However, the importance of PRMT1 in the regulation of cancer progression, and metastasis remains incompletely understood.

In the current study, we identified PRMT1 as a novel regulator of Epithelial-Mesenchymal-Transition (EMT), an essential process during cancer progression, and metastasis. Interestingly, the overexpression of PRMT1 in a non-transformed bronchial epithelial cell line resulted in the induction of EMT, characterized by a decrease in E-cadherin, and an increase in N-cadherin expression. Using a complementary approach, we also show that the gene silencing of PRMT1 in non-small cell lung cancer (NSCLC) cell lines lead to the reversal of EMT. Furthermore, PRMT1-mediated effects were not solely restricted to E- to N-cadherin switching. PRMT1 gene silencing in NSCLC cells also induced the formation of spheroids when cultured in Matrigel, and reduced migration and invasion, characteristics of epithelial cell phenotype. Moreover, we also identified Twist1, an important E-cadherin repressor, as a novel PRMT1 substrate and PRMT1-mediated methylation of Twist1 at arginine 34 (Arg-34) as an important event for E-cadherin repression. Thus, PRMT1 is shown to be a novel regulator of EMT and PRMT1 methylation of Twist1 at arginine 34 (Arg-34) as a unique “methyl arginine mark” for active E-cadherin repression.

## Experimental Procedures

### 

#### 

##### Constructs

Mouse Twist1 was obtained from Addgene (plasmid number 1783) and was engineered in-house into pCMV-Myc and pGEX-4T1 vectors. Site-directed mutagenesis was performed using QuickChange Site-directed Mutagenesis kit (Stratagene). Human PRMT1 cDNA sequence was subcloned into pCMV-HA vectors in-frame with HA tag sequence.

##### Cell Culture

Human non-transformed bronchial epithelial cell line (Beas2B), NSCLC cell lines (A549, H2122), and human breast cancer cell line (MCF7) were obtained from the tissue culture core of the University of Colorado, Anschutz Medical Campus. Beas2B, A549, and H2122 were cultured in RPMI medium supplemented with 10% FBS, in a humidified 5% CO_2_ incubator at 37 °C. Whereas MCF7 cells were cultured in DMEM medium supplemented with 10% FBS, in a humidified 5% CO_2_ incubator at 37 °C. All the cell lines were cultured bi-weekly and stocks of cell lines were passaged no more than ten times for use in experiments.

For generating A549 and H2122 clones with stable expression of non-targeting shRNAs and PRMT1 shRNAs, A549 and H2122 cells were transfected with either pENTR/H1/TO-control shRNA or pENTR/H1/TO-PRMT1 shRNA vectors followed by 200 μg/ml and 100 μg/ml Zeocin selection, respectively. Several zeocin-resistant clones were subsequently isolated and screened for PRMT1 knockdown via immunoblotting.

##### Three-dimensional Cell Culture

H2122 clones were grown in growth factor reduced Matrigel (BD Bioscience) basement membrane according to Debnath *et al.*, (18). Briefly, 5000 cells/well were grown in 2% Matrigel basement membrane with EGF on a 50% Matrigel layer. After 5–8 days pictures of the colonies were taken using an inverted microscope equipped with a digital camera. Images were later analyzed by determining the number of spheroids and aggregates and the spheroids/aggregates ratio is represented in the figure.

##### Wound Healing Assay (Scratch Assay)

Cells were grown to confluence in complete cell culture medium. At time 0, a 3-mm scrape wound was created across the diameter with a pipette tip followed by extensive washes with medium to remove dead and floating cells. After adding complete medium supplemented with 1 μg/ml Mitomycin C, cell migration was recorded at 0, 12, 24, and 48 h. Images were captured using an inverted microscope equipped with a digital camera. Images were later analyzed by determining the distance between the cells on either side of the scratch overtime, and are represented in the figure as percent scratch closure.

##### Cell Migration and Invasion Assays

For assessing cell migration, 30,000 cells in serum free media were seeded into the transwell inserts (Corning) containing 8-μm permeable pores and allowed to migrate toward 10% FBS-containing medium. Later, the cells in the transwell inserts were removed, and the inserts were washed in PBS three times. The migrated cells on the bottom of the insert were fixed with 2% glutaraldehyde solution followed by crystal violet (1%) staining. After washing the inserts three times with PBS, the inserts were allowed to air dry and pictures were taken using an inverted microscope. Ten independent fields were counted for each transwell and the average number of cells/field were represented in the graphs. For assessing cell invasion, 30,000 cells in serum-free medium were seeded in the matrigel-coated transwell inserts (BD Bioscience). The cells were later processed similar to that of cell migration assay.

##### Anchorage-independent Growth

Soft agar assays were performed as described previously (19). Briefly, 5,000 cells were plated in duplicates in a 6-well plate in growth medium containing 0.3% noble agar. After 14 days, colonies were stained and visualized with nitroblue tetrazolium chloride.

##### Experimental Lung Metastasis Assay

Animal experiments were conducted in strict accordance with the recommendations in the Guide for the Care and Use of Laboratory Animals of the National Institutes of Health. The animal protocol (13–016) was approved by the Institutional Animal Care and Use Committee (IACUC). H2122 clones with stable expression of non-targeting shRNAs and PRMT1 shRNAs were detached from the culture dish, collected by centrifugation, and re-suspended in PBS. For the lung metastasis assay, cells (1 × 10^6^ in 100 μl of PBS) were injected into the later tail veins of athymic nude mice (Harlan Laboratories). Mice were sacrificed after 8 weeks, and lung tumors were counted. Later, lungs were inflated *in situ* with PBS: Tissue-Tek O.C.T. compound (50:50) by intra-tracheal intubation, flash frozen, and embedded in Tissue-Tek O.C.T. compound. Hematoxylin and Eosin (H&E) staining was performed on the lung sections, and stained sections were later scanned using Aperio Scanscope CS and its associated Spectrum® image Management and Analysis system.

##### In Vitro Methylation Assays

*In vitro* methylation assays were performed as described previously (12, 13, 20). Briefly, GST-Twist1 or its mutants (2 μg) were incubated with HA-affinity matrix containing bound PRMT1 and 1 μCi of *S*-adenosyl-l-[methyl-^3^H]methionine (NEN Radiochemicals, 250 μCi, 9.25 MBq), at 30 °C for 1 h. After 1 h, the reactions were stopped and separated on a SDS-PAGE gel. The separated proteins were later transferred to nitrocellulose membrane, amplified (Autofluor, National Diagnostics, 2 h), dried, and fluorography was performed.

##### Indirect Immunofluorescence

IIF studies were performed as described earlier (20). Briefly, A549/H2122 clones stably expressing PRMT1 shRNAs, A549, and MCF7 cells expressing pCMV-Myc, Myc-Twist1, or Myc-Twist1-R34K mutant were fixed and stained for E-cadherin, and Myc (Twist1) followed by confocal microscopy. Image acquisition was performed using a Zeiss LSM Meta 510 confocal microscope with a 63×/1.2 Water correction objective using the following settings; laser intensities (543 nm-100%, 488 nm-6.0%, 405 nm-5.0%), Filters (Red-LP-560, Green-505–550, Dapi-BP-420–480), pinhole (Red-124 μm, Green-112 μm, Blue-98 μm), and stack size 512 × 512.

##### Knock-down Protocol

Double-stranded RNAs (siRNAs) targeting human PRMT1 (5′-GACUUCACCAUCGACCUGGACUUCA-3′), and control siRNAs (5′-UCUGUGAUUUGAAAGACUAGCCAAG-3′) were procured from Invitrogen (Carlsbad, CA) and Santa Cruz Biotechnology (SC-41069), while siRNAs targeting human Twist (SC38604) and control siRNA (SC-37007) were obtained from Santa Cruz Biotechnology. NSCLC cells were treated with 50 nm siRNAs by using Lipofectamine 2000 reagent according to the manufacturer's protocol. For generating stable clones, shRNA constructs were made by cloning the above PRMT1 siRNA sequence oligos into pENTR/H1/TO plasmid (Life Technologies).

##### Immunoblot Analysis

Cell extracts were prepared in a lysis buffer (0.5% Triton X-100, 50 mm β-glycerophosphate, pH 7.20, 0.1 mm sodium vanadate, 2 mm MgCl_2_, 1 mm EGTA, 1 mm dithiothreitol, 2 μg/ml leupeptin, and 4 μg/ml aprotinin). The following antibodies were used for immunoblotting: anti-E-cadherin and anti-N-cadherin (BD Bioscience), anti-PRMT1 (Cell Signaling Technologies), anti-Myc and anti-actin (Sigma Aldrich), and anti-HA (Roche Life Sciences). Aliquots of various extracts were resolved on 10–12% SDS-PAGE gels and transferred to nitrocellulose membranes. The filters were blocked in Tris-buffered saline (10 mm Tris-Cl, pH 7.4, 140 mm NaCl, containing 0.1% Tween 20 (TTBS) with 3% nonfat dry milk and then incubated with the same blocking solution containing the indicated antibodies at 0.5 μg/ml for 12–16 h. The filters were extensively washed in TTBS, and bound antibodies were visualized with horseradish peroxidase (HRP)-coupled secondary antibodies.

##### RNA Isolation and Real-time PCR

Total RNA from the cells were obtained using Trizol reagent (Invitrogen) as per the manufacturer's recommendations. For quantitative RT-PCR, 5 μg of total RNA was reverse transcribed using random primers and real-time PCRs were performed using the QuntiTect SYBR Green PCR kit (204050, Qiagen, Venlo, Limburg) and the Bio-Rad CFX real-time PCR detection system. The primers utilized in the PCR experiments were as follows; hPRMT1- F 5′-CCTTCACCTCCCCGTTCTG-3′, R 5′-CCAGGGCGTGCACGTAGT-3′, hTwist1-F 5′-GCGCTGCGGAAGATCATC-3′, R 5′-GGTCTGAATCTTGCTCAGCTTGT-3′.

##### Data Analysis

Data were compiled from at least three independent, replicate experiments, each performed on separate cultures and on separate occasions. The responses are displayed as “fold-changes.” Comparisons of data among experimental groups were performed using Student's *t* test for assessing variance. Increase in statistical significance (*p* value of <0.05) is denoted with an “*” symbol, while a decrease in statistical significance (*p* value of <0.05) is denoted with a “#” symbol.

## Results

### 

#### 

##### PRMT1 Is a Novel Regulator of EMT

**FIGURE 1.**
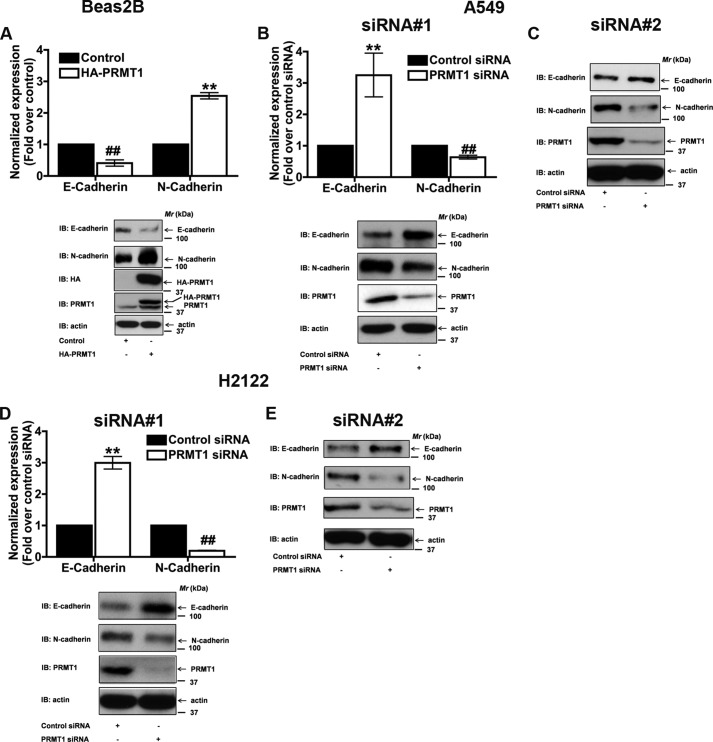
**PRMT1 is a novel regulator of EMT.** *A*, overexpression of PRMT1 in a non-transformed lung epithelial cell line (Beas2B) induced EMT, as detected by a decrease in E-cadherin and an increase in N-cadherin. Treatment of A549 (*B* and *C*) or H2122 (*D* and *E*) cells with two different PRMT1 siRNA sequences (siRNA#1 (Invitrogen), and siRNA#2 (Santa Cruz Biotechnology)) reversed EMT, as defined by an increase in E-cadherin and a decrease in N-cadherin. E-cadherin and N-cadherin protein levels were quantified by densitometry, normalized to their corresponding actin controls, and are represented in the figures as fold changes over control. **, *p* < 0.01; or ^##^, *p* < 0.01; *versus* control.

PRMT1 expression was shown to be up-regulated in cancers and important for cancer cell proliferation (16, 21). However, the role of PRMT1 in lung cancer progression and metastasis remains incompletely understood. Since, EMT is increasingly recognized as an essential event in cancer progression and metastasis, we evaluated first the role of PRMT1 on EMT. Two complimentary approaches were utilized to test our hypothesis. First, we determined the effects on EMT when PRMT1 was overexpressed in a non-transformed human bronchial epithelial cell line. Second, we determined the effects of PRMT1 gene silencing on EMT in NSCLC cell lines. To explore the role of PRMT1 overexpression on EMT, we made HA-tagged version of PRMT1 in a mammalian expression vector and employed in the transient transfections of a non-transformed lung epithelial cell line (*i.e.* Beas2B). The overexpression of PRMT1 was ascertained by immunoblotting with anti-PRMT1 and anti-HA antibodies (Fig. 1*A*). Interestingly, overexpression of PRMT1 induced a dramatic decrease in E-cadherin (Fig. 1*A*). Additionally, we also observed a remarkable increase in N-cadherin, a well-known mesenchymal cell marker (Fig. 1*A*). We next tested the effects of PRMT1 knockdown on EMT. For these studies, we designed and tested two different small interference RNA sequences (siRNAs) specific to PRMT1. Treatment of NSCLC cell lines (*i.e.* A549 and H2122) with PRMT1-specific siRNAs suppressed the expression of PRMT1 by more than 70% (Fig. 1, *B–E*). Notably, PRMT1 knockdown reversed EMT, as defined by an increase in E-cadherin expression and a decrease in N-cadherin expression (Fig. 1, *B–E*).

**FIGURE 2.**
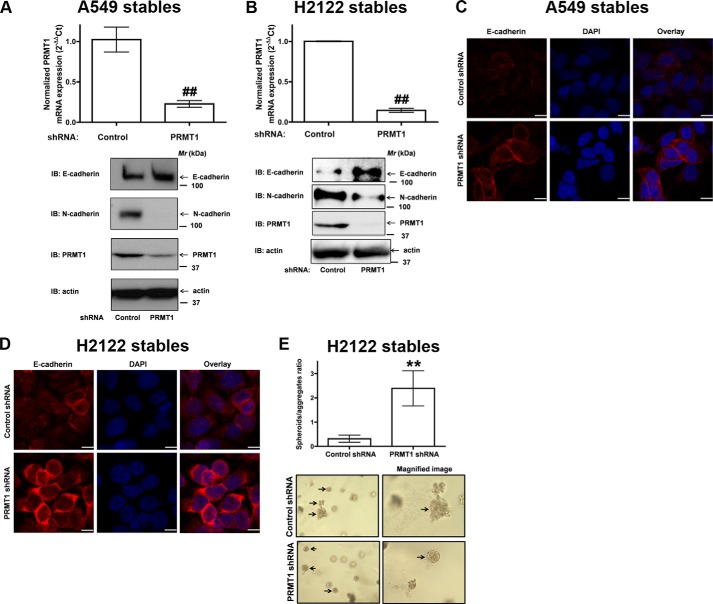
**PRMT1 knockdown induce epithelial phenotype in NSCLC cells.** A549 (*A*) or H2122 (*B*) clones with stable expression of control shRNA or PRMT1 shRNA were generated as described under “Experimental Procedures.” *Upper panel* represents qPCR analysis for PRMT1 knockdown efficiency, whereas *lower panels* represent immunoblots with E-cadherin, N-cadherin, PRMT1, and actin antibodies. ^##^, *p* < 0.01; *versus* control shRNA. *C* and *D*, A549, and H2122 clones stably expressing either control shRNA or PRMT1 shRNA were fixed, permeabilized and immunostained with anti-E-cadherin antibodies, and the expression of E-cadherin was visualized by indirect immunofluorescence and confocal microscopy. Scale bar 10 μm. *E*, H2122 clones stably expressing either control shRNA or PRMT1 shRNA were embedded in Matrigel as single cells as described under “Experimental Procedures.” The *upper panel* represents the ratio of spheroids/aggregates, whereas representative low power and magnified images are presented in the *lower panel*. **, *p* < 0.01; *versus* control shRNA.

Three-dimensional cell cultures are useful tools to examine the epithelial phenotype of cells wherein the epithelial cells would form spheroids (22, 23). To examine if PRMT1 knockdown in NSCLC cells results in a change in the epithelial phenotype of the cell, we generated A549 and H2122 clones with stable expression of non-targeting control shRNAs and PRMT1 targeting shRNAs (Fig. 2). Multiple stable clones were tested to exclude clonal variations. A549 and H2122 clones with stable expression of PRMT1 shRNAs showed significant knockdown of PRMT1 both at the mRNA and protein levels (Fig. 2, *A* and *B*). Consistent with the effects of PRMT1 specific siRNAs on EMT, multiple A549 and H2122 clones with stable knockdown of PRMT1 also showed an increase in E-cadherin expression as determined by Western blots (Fig. 2, *A* and *B*) and indirect immunofluorescence (Fig. 2, *C* and *D*). Furthermore, we also observed a striking decrease in N-cadherin expression (Fig. 2, *A* and *B*). The expression of EMT markers in stable cells (Fig. 2, *A* and *B*) is different from that of parental cell lines (Fig. 1, *B–D*), since the stable cell lines were homogenous as they were derived by single cell isolation technique. Interestingly, stable knockdown of endogenous PRMT1, in addition to augmenting E-cadherin expression, also induced the formation of spheroids when embedded in Matrigel matrices as single cells (Fig. 2*E*). Similar results were obtained with A549 clones stably expressing PRMT1 shRNAs (data not shown). Taken together, these data suggest that PRMT1 plays an important role in the regulation of E-cadherin.

##### PRMT1 Plays an Important Role in NSCLC Cell Migration, Invasion, and Metastasis

**FIGURE 3.**
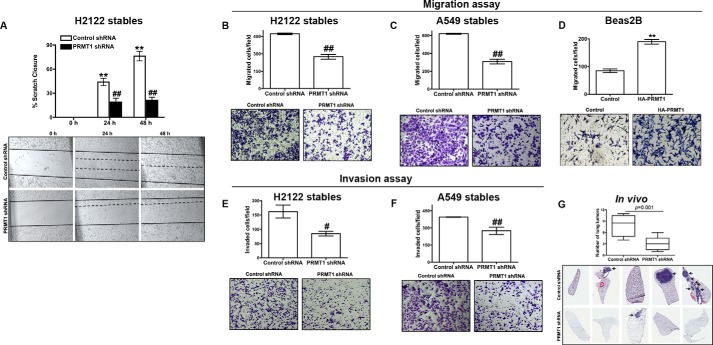
**PRMT1 knockdown results in reduced cell migration and invasion.** *A*, A 3-mm scrape wound was created in confluent cultures of H2122 clones with stable expression of either control shRNA or PRMT1 shRNA, and cell migration was recorded as described under “Experimental Procedures.” The *upper panel* represents the quantification of migration as described under “Experimental Procedures,” whereas representative images were presented in the *lower panel. B* and *C*, migration of H2122 (*B*) or A549 (*C*) clones with stable expression of either control shRNA or PRMT1 shRNA were assayed in trans-well inserts as described under “Experimental Procedures.” *Top panel* represents the number of cells migrated, whereas representative images are displayed in the *lower panel. D*, migration of Beas2B cells transiently transfected with empty vector or HA-tagged PRMT1 were assayed in trans-well inserts as described in the methods. *Top panel* represents the number of cells migrated, whereas representative images are displayed in the *lower panel*. **, *p* < 0.01; *versus* control. *E* and *F*, invasion of H2122 (*E*) or A549 (*F*) clones with stable expression of either control shRNA or PRMT1 shRNA were assayed in trans-well inserts coated with Matrigel as described under “Experimental Procedures.” *Top panel* represents the number of cells invaded, whereas representative images are displayed in the *lower panel*. ^##^, *p* < 0.01; ^#^, *p* < 0.05; *versus* control shRNA. *G*, H2122 clones stably expressing either control shRNA or PRMT1 shRNA were injected into athymic nude mice via tail-veins (*n* = 5). After 8 weeks, mice were sacrificed, visible tumors were counted and are represented in the graph, whereas representative H&E-stained lung sections were displayed in the *lower panel*.

We explored next if PRMT1 could also affect cell migration and invasion (Fig. 3). For testing the effects of PRMT1 knockdown on cell motility, we performed wound-healing assays (scratch assays, Fig. 3*A*). For these assays, a 3-mm scrape wound was created on confluent cultures of H2122 clones expressing either non-targeting shRNAs or PRMT1 shRNAs. The ability of the cells to migrate and heal the wound was recorded over a period of time in the presence of the mitosis blocker (Mitomycin C). H2122 clone expressing PRMT1 shRNAs displayed reduced motility in comparison to H2122 clone expressing non-targeting shRNAs (Fig. 3*A*). We have also tested the migratory potential of H2122 and A549 clones by using a trans-well assay (Fig. 3, *B* and *C*). For these assays, cells in serum free medium were seeded into the trans-well inserts and allowed to migrate through a permeable membrane (8 μm) toward 10% serum containing medium. The migrated cells on the bottom of the insert were later fixed, stained, counted and are represented in the graphs (Fig. 3, *B* and *C*). Interestingly, suppression of PRMT1 expression was associated with reduced cell migration in both A549 and H2122 clones (Fig. 3, *B* and *C*). Conversely, over expression of PRMT1 in a non-transformed bronchial epithelial cell line (Beas2B) was found to increase cell migration (Fig. 3*D*).

To measure the invasive abilities of A549 and H2122 clones expressing PRMT1 shRNAs, we performed invasion assays. In these assays, cells in serum free media were seeded into the trans-well inserts coated with a reconstituted basement membrane gel (Matrigel, BD Biosciences). The cells that have invaded the Matrigel and migrated through the pores were later fixed, stained, counted and are represented in the graphs (Fig. 3, *E* and *F*). A549 and H2122 clones expressing PRMT1 shRNAs also showed reduced invasion when compared with clones expressing non-targeting shRNAs (Fig. 3, *E* and *F*). These data suggest that PRMT1 is a novel regulator of cell migration and invasion.

To test the effects of PRMT1 knockdown on the metastatic potential of H2122 cells *in vivo*, H2122 cells stably expressing either PRMT1 shRNAs or non-targeting control shRNAs were injected into the lateral tail-veins of athymic nude mice, and the formation of lung tumors were examined. Consistent with the effects of PRMT1 knockdown on EMT, migration, and invasion, we observed a striking reduction in the number of lung tumors formed in mice injected with H2122 cells stably expressing PRMT1 shRNAs (Fig. 3*G*). We also confirmed that the effects of PRMT1 knockdown on the metatstatic potential of H2122 cells were not due to the induction of apoptotic cell death via AnnexinV/propidium iodide staining (data not shown). Thus, PRMT1 appears to act as an important regulator of tumor metastasis in lung cancer, and the effects of PRMT1 knockdown on tumor metastasis were not due to apoptotic cell death.

##### PRMT1 Is a Novel Regulator of Cell Transformation

**FIGURE 4.**
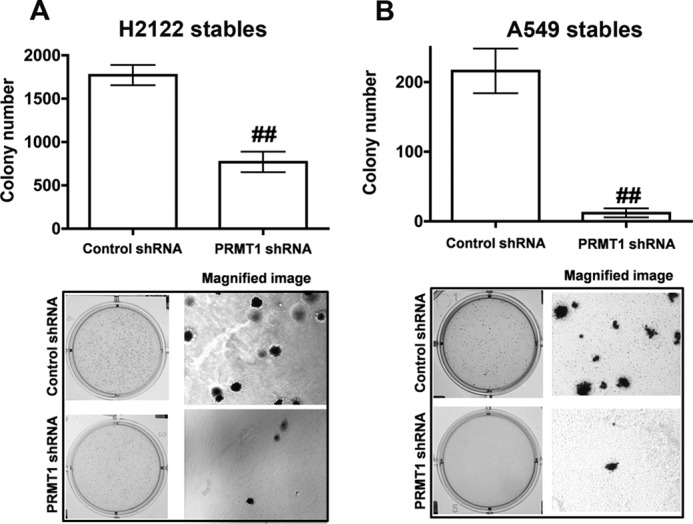
**PRMT1 regulates anchorage-independent growth.** Anchorage-independent growth of H2122 (*A*) or A549 (*B*) clones with stable expression of either control shRNA or PRMT1 shRNA were assayed using soft agar assays as described under “Experimental Procedures.” *Upper panel* represents the number of colonies formed, whereas representative low power, and magnified images are displayed in the *lower panel*. ^##^, *p* < 0.01; *versus* control shRNA.

The soft agar colony formation assay is a common method to measure anchorage-independent growth. To determine if PRMT1 also plays a role in transformed cell growth, A549 and H2122 clones stably expressing either PRMT1 shRNAs or non-targeting control shRNAs were seeded into soft agar cultures (Fig. 4, *A* and *B*). Remarkably, the knockdown of PRMT1 significantly attenuated colony formation of both A549 and H2122 clones expressing PRMT1 shRNAs compared with the clones with stable expression of non-targeting control shRNAs (Fig. 4, *A* and *B*). These results suggest that PRMT1, in addition to regulating the induction of EMT, cell migration, and invasion, also plays an important role in the transformation of cells.

##### Twist1, a Basic Helix-Loop-Helix Transcription Factor, Is a Novel PRMT Substrate

**FIGURE 5.**
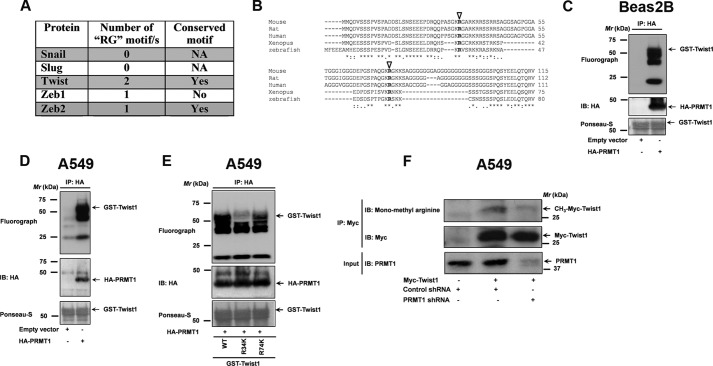
**Twist1 is a novel PRMT1 substrate.** *A*, *In silico* analysis of primary amino acid sequences of known E-cadherin repressors for PRMT methylation motifs. *B*, multiple sequence alignments of mouse, rat, human, *Xenopus*, and Zebrafish Twist1 amino acid sequences. Potential PRMT methylation sites are highlighted with *open arrowheads*. PRMT-mediated Twist1 methylation was evaluated by using an *in vitro* methylation reaction that includes HA-tagged PRMT1 purified either from Beas2B (*C*) or A549 (*D*) cell lysates, along with GST-Twist1, purified from *E. coli* and [^3^H]SAMe as a methyl donor as described under “Experimental Procedures.” The methylation status of Twist1 was revealed by SDS-PAGE followed by fluorography. After fluorography, the blots were stained with ponseau S to determine equal loading of the substrate (GST-Twist1, *lower panel*). *E*, PRMT1 purified from A549 cells were employed in an *in vitro* methylation reaction along with GST-Twist1 (wild-type) or its mutants R34K, and R74K and [^3^H]SAMe as a methyl donor. The methylation status of Twist1 and its mutants were later revealed by SDS-PAGE followed by fluorography. After fluorography, the blots were stained with Ponseau S to determine equal loading of the substrate (GST-Twist1 or its mutants, *lower panel*). *F*, to determine if Twist1 was methylated *in vivo*, A549 cells were co-transfected with Myc-tagged Twist1 and either control shRNA or PRMT1 shRNA. The lysates were later employed in immunoprecipiations with anti-myc antibodies. The methylation status of Twist1 was later determined by probing the blots with anti-monomethyl arginine-specific antibodies.

Since PRMT1 is a novel regulator of E-cadherin (Figs. 1 and 2), we explored if the family of E-cadherin repressors could serve as potential PRMT1 substrates. Interestingly, *in silico* analysis of primary amino acid sequences of known E-cadherin repressors (Snail, Slug, Twist1 and Zeb1/2) revealed Twist1 as a potential PRMT1 substrate (Fig. 5*A*). Twist1 displayed two potential PRMT methylation (RG) sites (Arg-34 and Arg-74, Fig. 5, *A* and *B*) that were well conserved across the species, suggesting that the arginine methylation sites might play an important role for Twist1 function (Fig. 5*B*).

To test directly if Twist1 was indeed a PRMT substrate, we performed an *in vitro* methylation assay (Fig. 5, *C* and *D*). For these assays, HA-tagged PRMT1 was expressed and purified from non-transformed bronchial epithelial cells (*i.e.* Beas2B, Fig. 5*C*) or NSCLC cells (*i.e.* A549, Fig. 5*D*). Recombinant Twist1 (GST-Twist1) was expressed and purified from *Escherichia coli*. The *in vitro* methylation reaction included the isolated PRMT1, GST-Twist1 and *S*-adenosyl l-methionine (SAMe) as a methyl donor. PRMT1 catalyzed strong methylation of Twist1 (Fig. 5, *C* and *D*).

Twist1 encodes two potential PRMT methylation sites (Arg-34 and Arg-74, Fig. 5, *A* and *B*). We next tested the specificity of PRMT1 to the predicted arginine residues (Arg-34 or Arg-74). For these experiments, we made methylation-deficient mutants of Twist1 by substituting arginines to similarly charged lysines (R34K, and R74K). Interestingly, PRMT1 efficiently catalyzed the methylation of wild-type and R74K mutant of Twist1 (Fig. 5*E*), but not the R34K mutant (Fig. 5*E*). Taken together, these results indicate that Twist1 is a novel PRMT1 substrate and PRMT1 predominantly catalyzes Arg-34 methylation of Twist1.

To determine if Twist1 was methylated endogenously (*in vivo*), we made use of methyl-arginine specific antibodies (Fig. 5*F* and Ref. 24). For these experiments, A549 cells were transiently transfected to express myc-tagged Twist1. Pull downs were performed on the cell lysates and the *in vivo* methylation status of the Twist1 was detected by immunoblotting with anti-mono methylarginine-specific antibodies (Fig. 5*F*). Pull downs performed on cells transfected with Myc-Twsit1 displayed methylation of Twist1 (Fig. 5*F*). Pull downs performed on cells transfected with empty vector, on the contrary, failed to show methylation and served as negative control (Fig. 5*F*). Additionally, *in vivo* methylation of Twist1 was severely attenuated in the absence of PRMT1 expression, indicating the specificity of the anti-mono methyl-arginine specific antibodies (Fig. 5*F*).

##### Arg-34 Methylation of Twist1 Is Required for E-cadherin Repression

**FIGURE 6.**
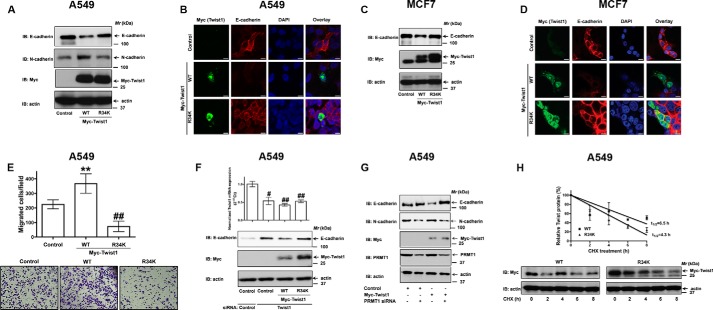
**PRMT-mediated Twist1 methylation is required for E-cadherin repression.** *A*, A549 cells were transiently transfected with either Myc-tagged wild-type or R34K mutant of Twist1. The lysates were later probed for the expression of E-cadherin and N-cadherin via immunoblotting with specific antibodies. *B*, A549 cells transfected with empty vector, Myc-tagged wild-type or R34K mutant of Twist1 were fixed, permeabilized, and co-immunostained with anti-myc antibodies and anti-E-cadherin antibodies, and the expressions of E-cadherin and Twist1 (Myc) were visualized by indirect immunofluorescence and confocal microscopy. Scale bar 5 μm. *C*, MCF7 cells were transiently transfected with either Myc-tagged wild-type or R34K mutant of Twist1. The lysates were later probed for the expression of E-cadherin and N-cadherin via immunoblotting with specific antibodies. *D*, MCF7 cells transfected with empty vector, Myc-tagged wild-type or R34K mutant of Twist1 were fixed, permeabilized and co-immunostained with anti-myc antibodies and anti-E-cadherin antibodies, and the expressions of E-cadherin and Twist1 (Myc) were visualized by indirect immunofluorescence and confocal microscopy. Scale bar 5 μm. *E*, migration of A549 cells transfected with empty vector, Myc-tagged wild-type or R34K mutant of Twist1 were determined using trans-well assays as described under “Experimental Procedures.” *Top panel* represents the number of cells migrated, whereas representative images are displayed in the lower panel. ^##^, *p* < 0.01; **, *p* < 0.01; *versus* control. *F*, A549 cells were co-transfected with Twist1 siRNA and either wild-type or R34K mutant of Twist1. The lysates were later probed for the expression of E-cadherin, Myc-Twist1 with specific antibodies. *G*, A549 cells were co-transfected with Myc-tagged Twist1 and either control siRNA or PRMT1 siRNA. Total lysates were later probed for the expression of E-cadherin, N-cadherin, PRMT1, and Twist1 (Myc). *H*, A549 cells transiently transfected with either Myc-tagged Twist1 or R34K mutant were treated with cycloheximide (100 μg/ml) for indicated periods of time, followed by immunoblotting and densitometric scanning. *Upper panel* represents the normalized Twist1 (Myc)/actin levels, whereas representative images are displayed in the *lower panel*.

Since Twist1 is methylated by PRMT1 specifically at arginine 34 (Arg-34, Fig. 5), we sought to interrogate the functional significance of R34 methylation of Twist1 on E-cadherin repression. For these experiments, we generated myc-epitope tagged versions of wild-type and R34K mutant of Twist1 for mammalian expression (Fig. 6). Expression of wild-type Twist1, as expected, repressed E-cadherin expression (Fig. 6*A*). Expression of R34K mutant, in contrast, failed to impact E-cadherin expression (Fig. 6*A*), suggesting that the Arg-34 methylation of Twist1 is required for E-cadherin repression. In addition, we also observed a corresponding increase in N-cadherin, a mesenchymal cell marker, by the wild-type Twist1 but not by the R34K mutant of Twist1 (Fig. 6*A*). Similar effects of R34K mutant of Twist1 on E-cadherin repression were also observed in H2122 cells (data not shown). We also confirmed the effects of Twist1 expression on E-cadherin repression via indirect immunofluorescence (Fig. 6*B*). A549 cells expressing wild-type Twist1 (Fig. 6*B*, *Myc panel*) displayed reduced E-cadherin staining, while A549 cells expressing R34K mutant displayed positive staining for E-cadherin (Fig. 6*B*). To ensure that the Twist1 arginine methylation-mediated E-cadherin repression was not restricted to the epithelium of the lung, we also tested the effects of wild-type and methylation-deficient mutants of Twist1 on E-cadherin repression in another epithelial cell-derived cancer cell line, MCF7 (a human breast cancer cell line). Consistent with the effects of the Twist1 mutants on E-cadherin repression in NSCLC cells, expression of the R34K mutant also failed to repress E-cadherin in MCF7 cells, as determined by Western blot (Fig. 6*C*), and indirect immunofluorescence (Fig. 6*D*).

We next investigated the effects of Twist1 (WT and R34K mutant) expression on A549 cell migration using trans-well assays (Fig. 6*E*). Expression of wild-type Twist1 in A549 cells, but not the R34K mutant of Twist1, resulted in increased cell migration (Fig. 6*E*). These results indicate that the arginine methylation of Twist1 at Arg-34 might be important for Twist1-induced cell migration.

To confirm the requirement of Arg-34 methylation for Twist1 function, we also performed “rescue” experiments in the Twist1 depleted (knockdown) A549 cells, employing wild type and R34K mutant of mouse Twist1 (Fig. 6*F*). Knockdown of Twist1 provoked increased E-cadherin expression (Fig. 6*F*). While the expression of wild type Twist1 could rescue Twist1 function (as determined by reduced E-cadherin levels), expression of the R34K mutant, on the contrary, failed to repress E-cadherin expression (Fig. 6*F*). Furthermore, failure of Twist1 to repress E-cadherin in the absence of PRMT1 (Fig. 6*G*) demonstrates the importance of PRMT1 methylation of Twist1 for E-cadherin repression. Taken together, our results identified a previously unknown functional aspect of the conserved arginine residue within Twist1 in E-cadherin repression. These studies further demonstrate that Twist1 methylation at Arg-34 represents an important “methyl arginine mark” that defines active E-cadherin repression

##### Arginine Methylation Might Play a Potential Role in the Nuclear Import of Twist1

Several studies highlight the importance of post-translational modifications, predominantly phosphorylation, in regulating the protein stability of Twist1 (25–27). To test if Arg-34 methylation of Twist1 is required for protein stabilization of Twist1, A549 cells expressing myc-tagged wild-type or R34K mutant were treated with the protein synthesis inhibitor, cycloheximide, and harvested at various times (0, 2, 4, 6, and 8 h). Twist1 levels were recorded by immunoblotting from total lysates using anti-myc antibodies (Fig. 6*H*). The Twsit1 levels were normalized against actin (by making use of anti-actin antibodies, Fig. 6*H*). Wild-type Twist1 decayed with a half-life of ∼6.5 h (Fig. 6*H*). Interestingly, there is no significant difference in the decay rates of R34K mutant (Fig. 6*H*, half-life of ∼4.3 h). These results suggest that Arg-34 methylation might not play a role in stabilizing Twist1 protein.

It was shown earlier that arginine methylation of proteins can influence their sub-cellular localization (20). Interestingly, confocal microscopy of wild-type Twist1 expressing cells revealed predominant nuclear staining of Twist1 in A549 (Fig. 6*B*) and MCF7 (Fig. 6*D*) cells. Expression of R34K mutant of Twist1, on the contrary, was observed to be predominantly cytoplasmic in both A549 and MCF7 cells (Fig. 6, *B* and *D*). These data would suggest that Arg-34 methylation of Twist1 might have a potential role in the regulation of nuclear import of Twist1.

## Discussion

EMT is a complex phenomenon and an important driver of tumor invasion, tumor progression, and tumor metastasis. Loss of E-cadherin expression is considered as a key event during the induction of EMT (28, 29). In the current study, we identify a novel role for PRMT1 in the regulation of EMT, mechanistically via the Arg-34 methylation of the E-cadherin repressor, Twist1. In the present study, we show that PRMT1 is an important regulator of E-cadherin via Western blotting and indirect immunofluorescence (Figs. 1 and 2). Most importantly, we show that depletion of PRMT1 induced spheroid formation, a characteristic of epithelial cell phenotype (Fig. 2*E*). As evinced from the works of Bissell, Brugge, and others, transformed cells tend to grow in a disorganized (form aggregates) manner in basement membrane cultures, while non-transformed epithelial cells, on the contrary, establish apical-basal polarity and grow as spheroids (18, 19, 30–32).

Interestingly, among the known E-cadherin repressors (Snail, Slug, Twist1, and Zeb1/2), only Twist1 was identified as a PRMT1 substrate. Considering the importance of Twist1 during tumor progression, we strongly believe that our findings will have far-reaching implications in gaining a better understanding of tumor progression in general.

Twist1 belongs to a family of basic helix-loop-helix proteins (bHLH), which function as transcription factors (33). Most importantly, Twist1 is an important E-cadherin repressor (34–37), and the overexpression of Twist1 in several cancers have been well documented (38–41). In addition, Twist1 overexpression also indicate poor overall survival rate (42) and poor prognosis, in part, due to the associated loss of E-cadherin expression (43). In the present study, we have uncovered a new dimension in the regulation of Twist1 functions by PRMT1. We have identified Twist1 as a novel PRMT1 substrate. Furthermore, PRMT1-mediated Twist1 methylation occurred specifically at Arg-34, a well-conserved residue within Twist1 sequences of various species. Strikingly, arginine methylation motifs identified within the Twist1 were also observed in other members of bHLH transcription factors *e.g.* scleraxis, paraxis, and Hand2. Interestingly, paraxis is also shown to specifically bind E-box elements, like Twist1 (44). However, the arginine methylation status of paraxis, and the potential role of paraxis in regulating E-cadherin remain to be established. Taken together, in addition to Twist1, it is possible that protein arginine methylation might also regulate the function/s of other family members of bHLH transcription factors.

The role of phosphorylation on Twist1 function has been previously reported (25–27). It was shown earlier that phosphorylation of Twist1 by protein kinase B at Ser42 leads to the inhibition of DNA damage-induced p53 activity (27) and phosphorylation by mitogen-activated protein kinases (MAPKs) at Ser-68, on the contrary, was shown to be important for the stabilization of Twist1 and promotion of breast cancer cell aggressiveness (25). Although, Arg-34 methylation of Twist1 has no effect on protein stability (Fig. 6*H*), indirect immunofluorescence detection of Twist1 expression revealed a possible role for Arg-34 methylation of Twist1 in nuclear import (Fig. 6, *B* and *D*). Since, basic helix-loop-helix proteins are known to form homo- and heterodimers, we speculate that arginine methylation of Twist1 might trigger homo- and/or hetero-dimerization of Twist1 (45), which facilitate subsequent nuclear import. However, the molecular details remain to be discerned.

We also highlight the importance of PRMT1 for the induction of EMT (Figs. 1, 2, 3). However, the upstream regulator/s of PRMT1 catalytic activities in lung cancer remain to be identified. In this regard, the molecular details of the effects of transforming growth factorβ (TGFβ), hepatocyte growth factor (HGF), Wnts, and/or hypoxia, which were shown to regulate E-cadherin repressors (Twist, Zeb, Slug, and Snail), and EMT (19, 46, 47), on the activation of PRMT1 catalytic activities and subsequent Twist1 methylation also remains to be evaluated.

In summary, our study provides a strong evidence for the involvement of PRMT1 in the induction of EMT, cancer cell migration, invasion, and metastasis. Our study also identified an important role for PRMT-mediated Twist1 methylation on E-cadherin repression, presenting PRMT1-mediated Twist1 methylation as a novel therapeutic target for developing more effective anti-invasive/anti-metastatic drugs. Methylated Twist1 (Arg-34), as such, could also emerge as a potential important biomarker for lung cancer. Thus, PRMT1 not only represents a unique class of EMT regulators but also represents a potential new class of drug targets for future therapeutic treatments of lung cancer.

## Footnotes

2 The abbreviations used are:

PRMT: protein arginine methyl transferase
RG: arginine-glycine-rich motifs
NSCLC: non-small cell lung cancer
SAMe: *S*-adenosyl l-methionine
EMT: Epithelial-Mesenchymal-Transition
H&E: Hematoxylin and Eosin
siRNA: small interference RNA
CDH: cadherin.
